# Myocardial scar and left ventricular ejection fraction classification for electrocardiography image using multi-task deep learning

**DOI:** 10.1038/s41598-024-58131-6

**Published:** 2024-03-29

**Authors:** Atirut Boribalburephan, Sukrit Treewaree, Noppawat Tantisiriwat, Ahthit Yindeengam, Titipat Achakulvisut, Rungroj Krittayaphong

**Affiliations:** 1 Department of Biomedical Engineering, Faculty of Engineering, Mahidol University, Nakhon Pathom, Thailand; 2 Looloo Technology, Bangkok, Thailand; 3 Division of Cardiology, Department of Medicine, Faculty of Medicine Siriraj Hospital, Mahidol University, 2 Wanglang Road, Bangkoknoi, Bangkok, 10700 Thailand; 4 Her Majesty Cardiac Center, Faculty of Medicine Siriraj Hospital, Mahidol University, Bangkok, Thailand

**Keywords:** Cardiology, Machine learning

## Abstract

Myocardial scar (MS) and left ventricular ejection fraction (LVEF) are vital cardiovascular parameters, conventionally determined using cardiac magnetic resonance (CMR). However, given the high cost and limited availability of CMR in resource-constrained settings, electrocardiograms (ECGs) are a cost-effective alternative. We developed computer vision-based multi-task deep learning models to analyze 12-lead ECG 2D images, predicting MS and LVEF < 50%. Our dataset comprises 14,052 ECGs with clinical features, utilizing ground truth labels from CMR. Our top-performing model achieved AUC values of 0.838 (95% CI 0.812–0.862) for MS and 0.939 (95% CI 0.921–0.954) for LVEF < 50% classification, outperforming cardiologists. Moreover, MS predictions in a prevalence-specific test dataset recorded an AUC of 0.812 (95% CI 0.810–0.814). Extracted 1D signals from ECG images yielded inferior performance, compared to the 2D approach. In conclusion, our results demonstrate the potential of computer-based MS and LVEF < 50% classification from ECG scan images in clinical screening offering a cost-effective alternative to CMR.

## Introduction

Coronary artery disease (CAD) is the leading cause of death and disability worldwide, with decreasing incidence in developed countries but increasing in developing countries^1^. CAD remains asymptomatic until the coronary stenosis becomes moderate or severe, resulting in the chest pain, dyspnea, and syncope^2^. However, approximately 30% of myocardial infarctions (MI) do not manifest with clear symptoms^3^. A missed MI diagnosis can lead to serious complications, such as left ventricular systolic dysfunction, heart failure (HF), and death.

HF was reported to affect 1–2% of the population^4,5^, and that rate is expected to increase over the next decade, posing a significant healthcare burden^6^. Left ventricular ejection fraction (LVEF) is used to classify HF patients for appropriate treatment^7,8^. Therefore, detection of MI and LVEF at an earlier stage can help clinicians make better decisions for further investigation and proposing an appropriate treatment^9^.

Cardiac magnetic resonance (CMR) imaging has the ability to accurately and non-invasively assess the heart's functional and anatomical abnormalities, including characterizing myocardial scars (MS), which is a common sequelae of MI^9,10^. Importantly, MS was reported to be one of the critical determinants, predicting the future development of heart failure^11^. However, CMR is expensive and requires highly trained personnel to perform imaging and interpretation, limiting its availability in remote or underdeveloped settings.

Electrocardiogram (ECG) is frequently employed as an initial investigation for diagnosing cardiovascular diseases due to its accessibility, whereas CMR is typically reserved for more in-depth investigations^12^. ECG scans contain patterns that can suggest cardiovascular diseases like CAD, MS, and left ventricular systolic dysfunction^13,14^. Trained cardiologists can identify these patterns, and diagnose the corresponding diseases. However, the availability of well-trained cardiologists is limited in remote or developing areas. Moreover, the interpretation of ECG scans is susceptible to both human error and interrater variability. Alternatively, computer-based ECG interpretation could help mitigate these limitations. Previous studies reported that machine learning systems can detect cardiovascular diseases, such as MI, arrhythmias, and left ventricular systolic dysfunction, using a 12-lead ECG^14–17^.

However, obtaining ECG data in machine-readable format (i.e., ECG tracings) can be challenging in resource-limited settings, including Thailand, since most ECG records are stored in paper or scanned format. Hence, computer image-based ECG classification for CAD detection may be advantageous in these circumstances.

In this paper, rather than using a separate classification model for each task, we introduce multi-task convolutional neural network (CNN) models that read 12-lead ECG scan images to identify both CAD scars and abnormal LVEF of less than 50% for clinical screening purposes. We provide our models as open-source software to improve CAD screening in resource-limited settings.

## Results

### Study population

**Figure 1:**
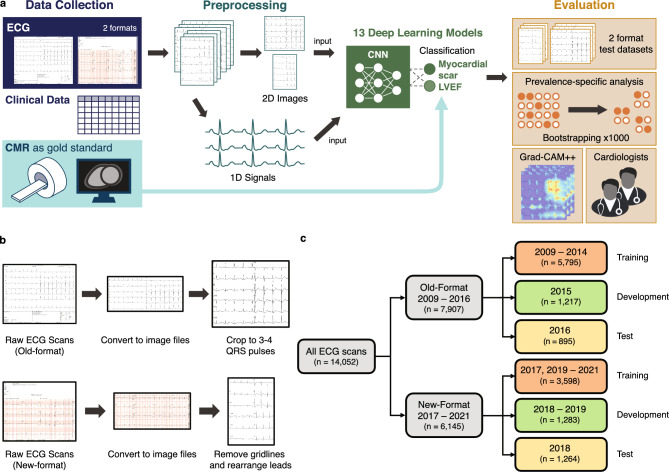
Horizontal flow diagrams describing the ECG classification framework. (**a**) Summary workflow of MS and LVEF classification from 12-lead ECG scans using a multi-task deep neural network. ECG and CMR were performed on the same date. Both 2D ECG images and 1D extracted signals were input to CNNs to classify MS and LVEF. Evaluation were done by using 2 format test dataset, prevalence-specific analysis, Grad-CAM++, and cardiologists. (**b**) Data preprocessing: The ECG in PDF format is converted to an image, the gridlines are removed, and the leads are rearranged so that they are in the same order on every image. (**c**) Data splitting: The old-format and new-format test datasets are both split by year into training, development, and test sets. *CMR* cardiac magnetic resonance, *CNN* convolutional neural network, *ECG* electrocardiogram, *LVEF* left ventricular ejection fraction, *MS* myocardial scar, *PDF* Portable Document Format.

A total of 13,707 patients and 14,826 ECGs were retrospectively enrolled in this study. To prevent cross-dataset contamination, 774 ECGs were excluded, resulting in a total of 14,052. The population comprises two ECG formats, specifically the non-grid (old) format and the grid (new) format ECGs, collected using different machines. The following baseline characteristics represented the total number of ECGs. The average age of all ECGs was 72.29 ± 13.81 years and 50.53% were acquired from male patients. The prevalence of MS and LVEF < 50% in the total ECGs was 27.11% and 18.72%, respectively, while 12.35% belonged to the LVEF < 40% group. A total of 10.04% of all ECGs had both subendocardial and transmural scarring. ECGs with only subendocardial scarring or only transmural scarring accounted for 9.45% and 7.61% of the population, respectively. Table 1 shows the baseline data of the overall ECG population and each dataset used in the study. Out of the total ECGs, 5,407 (38.48%) had no clinical feature data except for age and sex, while all other ECGs had complete clinical data. The missing data were imputed as described in the methods section. Summary workflow of MS and LVEF classification system is shown in Fig. 1a.

**Table 1:** Baseline data of the overall study population, and of those included in each dataset.

Data	Age (year) Mean ± SD	Male gender n (%)	Current/ex-smoker n (%)	HT n (%)	DM n (%)	DLP n (%)	LVEF < 50% n (%)	LVEF < 40% n (%)	MS n (%)	Subendocardial scar only n (%)	Transmural scar only n (%)	Subendocardial scar AND Transmural scar n (%)
Population (N = 14,052)	72.3 ± 13.8	7100 (50.5%)	3672 (26.1%)	10,673 (76.0%)	6303 (44.9%)	9646 (68.6%)	2630 (18.7%)	1736 (12.4%)	3809 (27.1%)	1328 (9.5%)	1070 (7.6%)	1411 (10.0%)
Training^a^ (N = 9393)	73.2 ± 13.8	4822 (51.3%)	2772 (29.5%)	7668 (81.6%)	4522 (48.1%)	7027 (74.8%)	1803 (19.2%)	1197 (12.7%)	2655 (28.2%)	962 (10.2%)	677 (7.2%)	1016 (10.8%)
Development^a^ (N = 2500)	70.5 ± 13.4	1182 (47.3%)	539 (21.6%)	1671 (66.8%)	1014 (40.56%)	1469 (58.8%)	426 (17.0%)	277 (11.1%)	589 (23.5%)	207 (8.3%)	186 (7.4%)	196 (7.8%)
Test (old format) (N = 895)	71.4 ± 13.8	455 (50.8%)	320 (35.8%)	781 (87.3%)	483 (54.0%)	704 (78.7%)	173 (19.3%)	109 (12.2%)	236 (26.3%)	72 (8.0%)	76 (8.5%)	88 (9.8%)
Test (new format) (N = 1264)	69.7 ± 13.9	641 (50.7%)	41 (3.2%)	553 (43.8%)	284 (22.5%)	446 (35.3%)	228 (18.0%)	153 (12.1%)	329 (26.0%)	87 (6.9%)	131 (10.3%)	111 (8.8%)

^a^Contains both old and new ECG format.

*DLP* dyslipidemia, *DM* diabetes mellitus, *ECG* electrocardiogram, *HT* hypertension, *LVEF* left ventricular ejection fraction, *MS* myocardial scar, *SD* standard deviation.

### Model performance

We trained eight deep learning models: (1) Multi-task both formats, (2) Multi-task old-format only, (3) Transferred multi-task model, (4) Single-task for MS classification (both formats), (5) Single-task for LVEF classification (both formats), (6) Multi-task with clinical features, (7) Single-task (MS) with clinical features, and (8) Single-task (LVEF) with clinical features.

**Figure 2:**
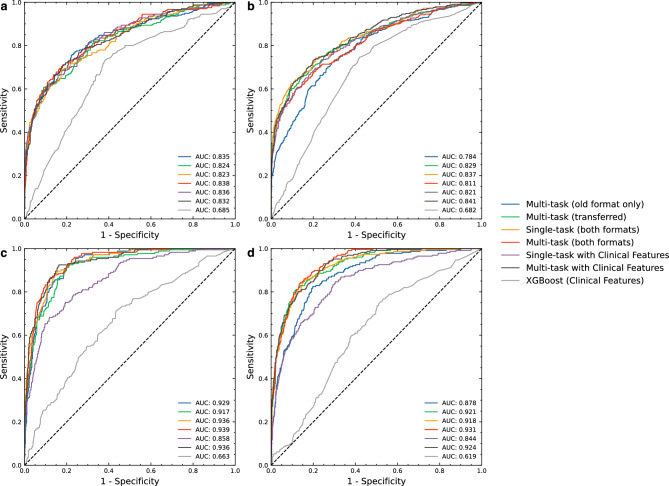
ROC curves of each evaluated 2D model. ROC curves showing the specificity and sensitivity of MS classification for each of the evaluated models using the old-format (**a**) and new-format (**b**) test sets; and of LVEF < 50% classification for each of the evaluated models using the old-format (**c**) and new-format (**d**) test sets. The AUC was quite similar between the old- and new-format test sets for all models for both MS and LVEF < 50%. *AUC* area under the ROC curve, *LVEF* left ventricular ejection fraction, *MS* myocardial scar, *ROC* receiver operating characteristic.

Overall, our multi-task both formats model outperformed the transferred multi-task model and the multi-task old-format only model in both MS classification and LVEF classification, except for MS classification on new-format test dataset. The AUCs for the multi-task both formats model for MS classification were 0.838 (95% CI 0.812–0.862) and 0.811 (95% CI 0.788–0.832) for the old-format and new-format test datasets, respectively. For detecting LVEF < 50%, the AUCs of the multi-task both formats model were 0.939 (95% CI 0.921–0.954) and 0.931 (95% CI 0.915–0.944) for the old-format and new-format test datasets, respectively (Fig. 2). Model performance results compared to baseline prediction are shown in the Supplementary Data 1.

Regarding the ECG interpretation performance of cardiologists on new-format test data, the AUCs for MS classification by an experienced cardiologist and an in-training cardiologist were 0.683 (95% CI 0.659–0.707) and 0.657 (95% CI 0.632–0.681), respectively. Both cardiologists had similar sensitivity (44.1% *vs.* 44.50%, respectively); however, the experienced cardiologist had a higher specificity (92.60%) than the in-training cardiologist (86.40%) (Supplementary Fig. 1).

**Figure 3:**
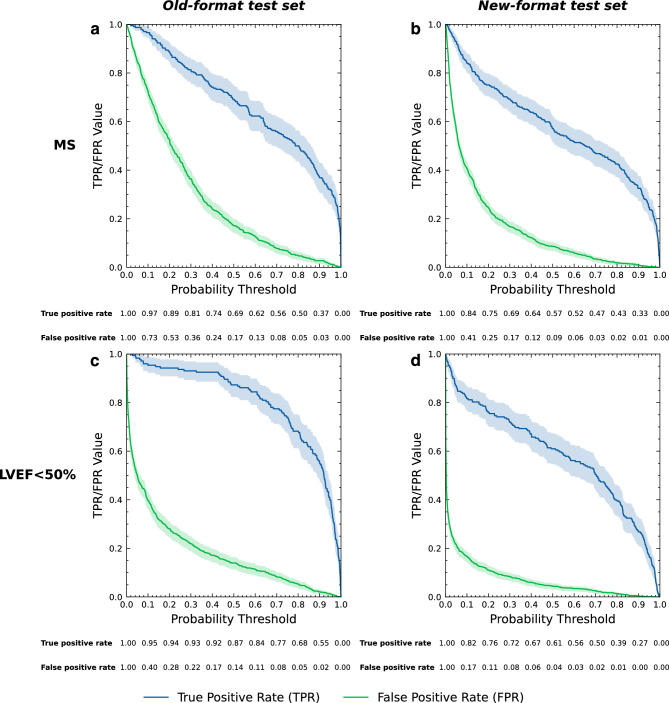
Classification plots of the 2D multi-task with clinical features model. The plots show the TPR and FPR value at various probability thresholds with 95%CI of MS classification when using the old-format (**a**) and new-format (**b**) test sets; and of LVEF < 50% classification when using the old-format (**c**) and new-format (**d**) test sets. *FPR* false positive rate, *LVEF* left ventricular ejection fraction, *MS* myocardial scar, *TPR* true positive rate, *95%CI* 95% confidence interval.

All of our developed models that were designed to evaluate ECG images as input features outperformed the XGBoost model, which was designed to evaluate only standard clinical features, by up to 50.40% (Fig. 2). The multi-task with clinical features model was able to classify MS in the old-format test dataset with specificity of 66.92%, 44.61%, and 30.50% at 80.00%, 90.00%, and 95.00% sensitivity, respectively. For LVEF < 50% classification when using the old-format test dataset, the model achieved the specificity of 90.03%, 84.76%, and 66.90% at 80.00%, 90.00%, and 95.00% sensitivity, respectively (Fig. 3).

### Incorporating clinical features

Incorporating clinical features into our multi-task models resulted in improved model performance in some cases (Fig. 2). For the single-task model, adding clinical features provided a performance boost only in MS classification in old-format test datasets. For the multi-task model, the performance boost was observed only in MS classification in new-format test datasets. The multi-task with clinical features model greatly outperformed the XGBoost model (which used only clinical features) with an AUC of 0.841 (95% CI 0.819–0.860) compared to 0.682 (95% CI 0.655–0.707) in MS classification using the new-format test dataset. (Fig. 2).

### Prevalence-specific analysis

When using the prevalence-specific test dataset, our multi-task both formats model achieved an AUC for MS classification of 0.812 (95% CI 0.810–0.814) and an F1-score of 0.931 (95% CI 0.931–0.932).

### Performance in detecting LVEF < 40%

Our sensitivity analysis showed similar performance among models when comparing LVEF < 50% detection and LVEF < 40% detection. The multi-task model with clinical features achieved the highest AUC of 0.942 (95% CI 0.925–0.956) when using the old-format test dataset, while the multi-task model achieved the highest AUC of 0.939 (95% CI 0.924–0.951) when using the new-format test dataset (Supplementary Fig. 2).

### Localization of models’ decision

**Figure 4:**
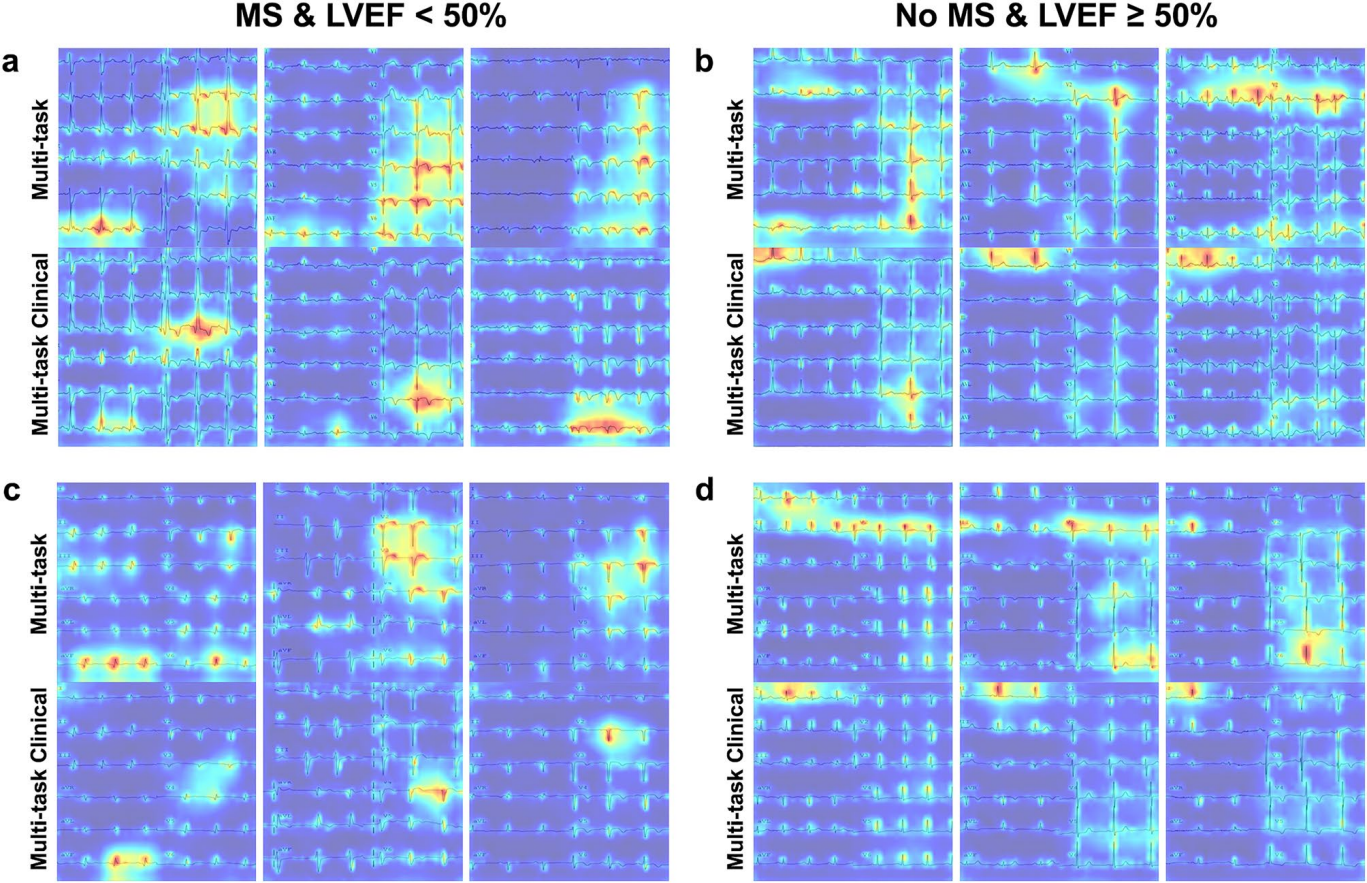
Examples of “Grad-CAM++” technique applied to ECG images. The “Grad-CAM++” technique for deep convolutional networks applied to ECG images highlighting areas that contributes to the prediction of Multi-task both format model and Multi-task with Clinical features model in (**a**) 3 cases of MS and LVEF < 50% from old-format test dataset. (**b**) 3 cases of No MS and LVEF ≥ 50% from old-format test dataset. (**c**) 3 cases of MS and LVEF < 50% from new-format test dataset. (**d**) 3 cases of No MS and LVEF ≥ 50% from new-format test dataset. The heatmaps were generated using PyTorch library for CAM methods^47^. *ECG* Electrocardiogram, *LVEF* left ventricular ejection fraction, *MS* myocardial scar.

We applied Grad-CAM++ to visualize the areas of ECG images that influenced the model decision. Figure 4 shows examples of heatmaps generated on top of the ECGs for multi-task and multi-task with clinical model. The heatmap highlighted the area with ECG tracings with greater emphasis on the area associated with models’ decisions. In cases with MS and LVEF < 50%, we observed that the model focused on abnormal Q waves, QRS complexes, and T wave inversions. For cases with no MS and LVEF ≥ 50%, the multi-task model generally highlighted QRS complexes in lead I, II, V2, and V6, while the multi-task with clinical model focused on lead I. Interestingly, the multi-task with clinical model appears to focus more on fewer leads compared to the multi-task model.

### 1D signal extraction and 1D model performance

We extracted 1-Dimension (1D) signals from the ECG images and trained five 1D deep learning models: (1) Multi-task both formats, (2) Multi-task old-format only, (3) Transferred multi-task model, (4) Single-task for MS classification (both formats), (5) Single-task for LVEF classification (both formats).

**Figure 5:**
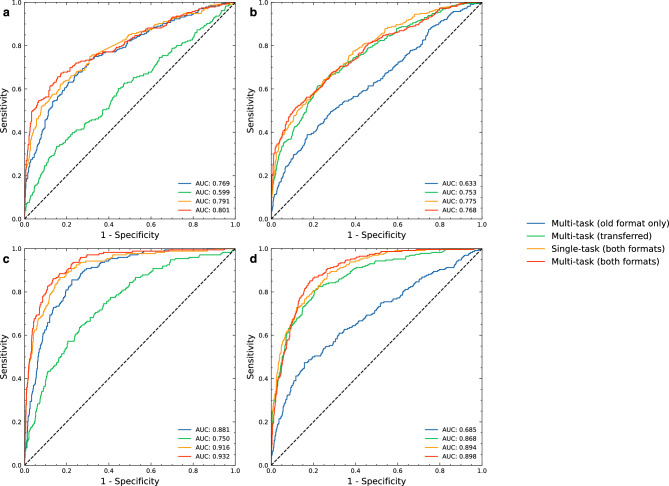
ROC curves of each evaluated 1D model. ROC curves showing the specificity and sensitivity of MS classification for each of the 1D models using the old-format (**a**) and new-format (**b**) test sets; and of LVEF < 50% classification for each of the evaluated models using the old-format (**c**) and new-format (**d**) test sets. *AUC* area under the ROC curve, *LVEF* left ventricular ejection fraction, *MS* myocardial scar, *ROC* receiver operating characteristic.

The results consistently demonstrate the superior performance of our 2D CNN models over their 1D counterparts in both MS and LVEF range classification tasks (Figs. 2, 5, and Supplementary data 2). Among the 1D models, our multi-task model stands out, maintaining the highest overall performance for both tasks.

## Discussion

In this study, we demonstrated that a multi-task deep learning model could detect MS and classify the LVEF range using a 12-lead ECG scan image. Our top-performing models, the multi-task both formats model and multi-task model with clinical features, demonstrated high performance in detecting MS and LVEF < 50% in both old and new ECG formats. They consistently exhibited comparable or superior performance when compared to their single-task counterparts in the majority of scenarios. Additionally, our model also achieved a high AUC and F1-score in MS prediction from the prevalence-matched population. The F1 score achieved in this prevalence-matched population was notably higher than that in our test sets, where the prevalence exceeded 26%. This finding might suggest that the model performs better in populations with lower prevalence, which more closely resemble real-world populations.

Given the variation in ECG format among different machines, it becomes crucial for the ECG image classification model to acquire visual features that can be universally applied across these formats. Our findings reveal that amalgamating ECG scans from various formats into a unified training dataset resulted in improved performance compared to segregating datasets based on ECG format. Additionally, the multi-task model designed for the old ECG formats also exhibited commendable performance when tested with a new-format dataset, underscoring its efficacy in predicting ECGs with diverse formats. However, a preprocessing protocol is needed to automatically prepare the ECG image prior to interpretation by the model.

The multi-task model also has a computational advantage since it shares the same backbone for predicting MS and LVEF. Several studies demonstrated performance gains in using a multi-task model in medical image analysis^18^. In addition to the computational edge, the multi-task model may also have a clinical advantage. When determining whether a patient has MS, the ability to simultaneously predict LVEF range might provide the impact of the scar on cardiac function. Likewise, when predicting LVEF < 50%, the model could suggest the etiology of impaired cardiac pumping by detecting the ischemic scar. Taken together, the advantages of our developed multi-task deep learning model suggest the strong potential of its use as a screening tool for MS and LVEF < 50% from ECG scan images in limited-resource settings.

Previous studies have demonstrated the potential of deep learning for MS detection, mainly focusing on raw ECG traces^19,20^. A similar study achieved a comparable AUC to our model, with CMR as the gold standard^20^. Another study utilizing data from CMR-confirmed ECGs and a publicly available ECG dataset without CMR measurements, reported a model with superior performance to our model. However, their model combines vectorcardiography with ECG for prediction^19^. We hypothesize that vectorcardiography may provide more detailed heart conduction information than ECG alone.

The PhysioNet in Cardiology Challenge 2020 presented a large multi-institutional database of ECG signals with remarkable results, utilizing deep learning and CNN^21^. As we have no access to the raw ECG traces, we extracted the signals from the ECG images and trained 1D CNN models. The results show that the models trained on ECG images perform better than the ones trained on the extracted signals; these results align with previous research study^22^.

In addition to performance differences, an intriguing distinction emerged between the 1D and 2D multi-task models during our transfer learning experiment. Notably, when assessing the 1D multi-task (old-format only) model on the new-format test set, we observed more pronounced performance degradation compared to its 2D counterpart. Moreover, the 1D multi-task (transferred) model exhibited poorer performance on the old-format test set. Overall, the findings suggest that 2D CNN models may exhibit better generalization to unseen formats (i.e., trained on old-format data and tested on new-format data) and retain previous knowledge more effectively (i.e., avoiding forgetting old-format data).

Nevertheless, the 1D model remains viable due to the potential to utilize a pretrained model from extensive 1D ECG databases in a range of cardiovascular diseases, improving generalizability in real-world applications^23^. However, the precision mismatch between raw traces in public 1D databases and image-extracted signals presents a potential hurdle for knowledge transfer to specific use cases. This aspect requires further investigation in future studies.

Numerous methodologies have been developed for the automated identification of MI from ECGs, ranging from simple thresholding techniques to sophisticated deep learning models^24,25^. Another study used ECG images annotated by cardiologists as input for MI detection using deep learning^26^. In the present study, we utilized DE-CMR as the true label, resulting in higher sensitivity for detecting MS cases, capturing more subtle signs and less severe MS cases. Notably, the reported model performance may vary among studies due to differences in the gold standard used and the prevalence of MS in the test dataset.

Observational studies showed that characteristics of ECGs are associated with LVEF^27,28^. Many recent studies also applied deep learning models to LVEF range classification using 2D ECG images^17,29–31^. Two of those previous studies assembled a larger ECG image dataset (compared to the size of our training dataset) for model training, and they measured LVEF using transthoracic echocardiography^17,30^. Despite using less training data, we still achieved comparable performance of LVEF prediction, which may suggest the effectiveness of our multi-task backbone for evaluating ECG images.

We found that adding clinical features as an input to our multi-task model could enhance its performance in MS classification in some test datasets. However, adding clinical features slightly reduced the LVEF < 50% classification performance. Additional study is needed to better understand the relationship between clinical features and LVEF. Interestingly, our multi-task both formats model outperformed cardiologist performance of ECG interpretation without the clinical features. This suggests that our model may have learned subtle patterns from ECGs that are unnoticeable by cardiologists.

We introduce our model as open-source software to provide a tool that could improve CAD screening in resource-limited settings. As our models are image classification models, our models could be deployed as a web application that allows taking photos of ECG by a smartphone, which does not require the investment in raw-ECG tracings data acquisition and storage and can easily integrate into hospital workflow.

### Strengths and limitations

To our knowledge, this is the first study to demonstrate the potential of multi-task deep learning models for classifying ECG images with corresponding same-date CMR as the gold standard for identifying the MS and the LVEF range. Same-date CMR provides a concurrent and precise comparison between ECG image and CMR assessment of MS and LVEF. Moreover, evaluation of the old-format model on new-format data gives us some positive indications of its performance on the unseen ECG format. However, further studies are needed to assess the performance of our multi-task model when using other ECG formats. Our study also has some mentionable limitations. Our prediction model requires semi-manual preprocessing of the ECG images before being fed into the model, which are ECG lead position identification in the image, ECG lead arrangement, and adjusting the number of QRS pulses. Once the ECG lead position and arrangement data are entered, unseen ECG formats can be easily integrated into the pipeline. Furthermore, our study only enrolled cases with corresponding same-date CMR imaging to ensure that MS cases were correctly classified; therefore, the number of enrolled cases is limited.

## Methods

### Study population

We retrospectively collected 12-lead ECG data from adult patients aged ≥ 18 years with suspected CAD or a history of CAD who underwent CMR imaging at the Faculty of Medicine Siriraj Hospital, Mahidol University from 2009 to 2021. Every enrolled participant underwent an ECG scan on the same date and prior to CMR, during which expert cardiologists interpreted the characteristics of MS, and determined the LVEF. MS cases that did not satisfy the criteria for MS caused by MI were excluded from this study^32,33^. Several patients underwent multiple CMR imaging sessions at different visits, and therefore, we evaluated each session as a distinct case. As a result, the number of included ECGs exceeded the number of patients enrolled in this study. The protocol for this study was approved by the Siriraj Institutional Review Board (SIRB), in accordance with the Declaration of Helsinki (MU-MOU CoA no. 469/2022). The need for informed consent was waived by the ethics committee of Siriraj Hospital, Mahidol University.

### Cardiac magnetic resonance imaging protocol

The CMR protocol included black blood axial plain images and steady-state free precession (SSFP) cine images of standard long-axis, 2-chamber, 3-chamber, and 4-chamber views. LVEF was assessed. Delayed-enhancement cardiovascular magnetic resonance (DE-CMR) images were interpreted to determine the location and pattern of MS. CMR and DE-CMR parameters are detailed in the Supplementary Methods. CAD scar was diagnosed using DE-CMR when the hyper-enhanced area was transmural or subendocardial pattern—either of which indicates MI^32,33^.

### Data collection and processing

The ECG scan images were stored in Portable Document Format (PDF). Our center has two different ECG acquisition systems, resulting in two different ECG formats—namely, the non-grid (‘old’) format (GE MAC 1200; GE Marquette Medical Systems, Milwaukee, WI, USA) and the grid (‘new’) format (Philips PageWriter TC30; Philips Healthcare, Best, the Netherlands). We decided to include both ECG formats to obtain a larger dataset for training, and to develop models that can read different ECG formats. CMR interpretation and clinical features data were stored in tabular format.

We transformed raw PDFs of full ECG reports into 12-lead ECG scan images through a three-step preprocessing procedure (Fig. 1b). Full details of the preprocessing steps are in the Supplementary Methods.

The overall dataset was divided into training, development, and test sets according to the year of ECG acquisition (Fig. 1c). The details are in the Supplementary Methods. Some patients in our study underwent more than one CMR session over the study period. To prevent training, development, and test dataset contamination, we exclude the CMR sessions from the same patient that belonged to more than one dataset, prioritizing the number of ECGs in the training dataset. The old-format and new-format test sets were kept separate for a proper evaluation of the use of different ECG formats. All data underwent de-identification to remove any patient-specific information.

### Clinical features

We also collected six risk factors commonly used to identify CAD risk in clinical practice, including age, sex, and histories of smoking (current or ex-smoker), diabetes mellitus, hypertension, and dyslipidemia. Age was the only numerical variable, while the rest were categorical variables.

Some clinical feature data were unavailable for some patients due to the retrospective nature of our study. Our solution was to employ a multivariate imputation method to impute the missing data^34^. In this study, each feature with missing values was modeled as a function of age and sex, which were available in all patients. Bayesian Ridge regression^35^ was employed as the function estimator. We solely applied imputation to the training dataset to maintain fairness during evaluation, leaving the data in the test sets unchanged.

### 1D signal extraction from images

We performed a 1D signal extraction from 2D images by extracting the pixels for each lead. Here, we exclude 5 training samples due to overlapped signals and signals overlap with the text. After extraction, some y-axis pixels (voltage axis) may correspond to the same x-axis pixels (time axis). We use a simple preprocessing to average the voltage for duplicated time pixels. Additionally, we interpolate the missing extracted data using the next available values. Then, we align all extracted signals using Christov R-peak detection and crop to the same window size of − 0.5 to + 1.5 s^36,37^.

### Model development

We aimed to create a deep learning model that can classify MS and the LVEF range using an ECG scan image. We selected an LVEF cut point of < 50% since HF patients with LVEF < 50% are managed differently than patients with LVEF ≥ 50% (preserved ejection fraction)^8^.

**Figure 6:**
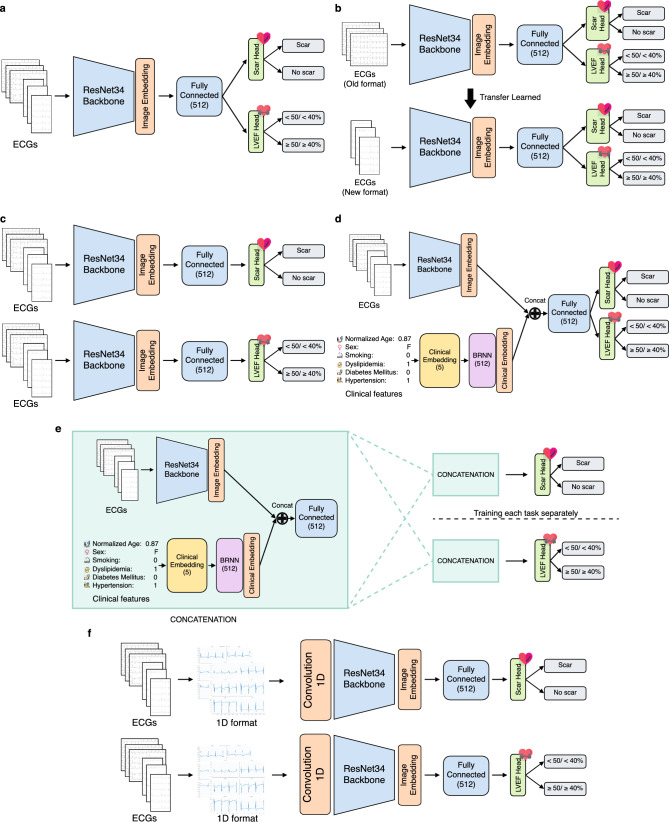
Model architecture of five different training paradigms. (**a**) Multi-task model: This model combines old- and new-format datasets to train multi-task MS and LVEF range classification. (**b**) Transferred multi-task model: This model is pretrained using old-format ECG data and then fine-tuned using new-format ECG data. (**c**) Single-task model: In this model, the old and new ECG formats are combined to train the MS model and LVEF model separately. (**d**) Multi-task model with clinical features: This model combines clinical features with ECG image data to predict MS and LVEF range. (**e**) Single-task model with clinical features: This model combines clinical features with ECG image data and was trained separately to predict MS and LVEF. (**f**) 1D Single-task model: 1D ECG signals are extracted from the images to train MS model and LVEF model separately. *1D* 1 Dimension, *BRNN* bidirectional recurrent neural network, *ECG* electrocardiogram, *LVEF* left ventricular ejection fraction, *MS* myocardial scar, *RNN* recurrent neural network.

We propose a multi-task ECG classification model that uses a neural network with a shared backbone for image feature extraction, and layers for multi-task MS and LVEF classification (Fig. 6). The model architecture is specified in the Supplementary Methods. We choose ResNet-like algorithms as the foundational framework for our model, drawing inspiration from their proven success in diverse image classification tasks, for example, ImageNet^38^. Furthermore, existing research attests to the effectiveness of ResNet-like algorithms in 1D ECG classification tasks^39–41^. Since we had both old and new ECG datasets, the model was trained under different training paradigms to compare the performance of the model between the two datasets. We propose three training paradigms and a baseline for comparison.

#### Multi-task MS and LVEF classification

In this paradigm, we combined the old- and new-format training and development datasets to train a multi-task model. In multi-task learning, we weighed the cross-entropy loss ratio between MS and LVEF predictions equally (Fig. 6a). However, we also experimented with other cross-entropy loss ratios including 60:40 and 70:30. Doing so makes the loss contribution from scar predictions more significant. Therefore, the model learns to prioritize MS prediction over LVEF (Supplementary Data 3).

#### Transferred multi-task MS and LVEF classification

Regarding visual features, the old- and new-format ECG records could be too distinguished from each other when perceived by the model so the model could not learn from both formats simultaneously. Thus, this paradigm divides the training process into two steps. First, the pretrained model used only the old-format data for pretraining. We then fine-tuned the model using new-format ECGs (Fig. 6b).

#### Single-task MS and LVEF classification

The single-task model was trained to predict MS and LVEF separately (Fig. 6c). This paradigm helps to verify if multi-task training helps improve classification performance. We combined the data from the old and new formats for model training, with cross-entropy loss.

#### Baseline

A baseline prediction is the most greatly represented class for each task. In our models, our baseline predictions are no MS and LVEF ≥ 50%.

### Incorporating clinical features

To validate whether the multi-task model can be enhanced via the addition of clinical features, we trained a model using both ECG scan images and patient clinical features (Fig. 6d,e). Comprehensive details of the model are in the Supplementary Methods.

We also trained an eXtreme Gradient Boosting (XGBoost)^42^ model with only the clinical features to compare the models. This model represents the predictive performance of the CAD screening approach without using ECG images.

### 1D models

We used a similar ResNet architecture for our 1D CNN which resembles the architecture of our 2D CNN model. The structures are nearly identical, except for the first layer, where we replace the 2D convolution with a 1D convolution (Fig. 6f). However, unlike the 2D model, the 1D version handles 12 lead signals instead of a single ECG image. We vectorize the lead signals and input them individually to the ResNet with shared weights. Finally, we average the embeddings from the 12 leads and pass them to the prediction head. Similar to our image-based experiments, we trained the model using three training paradigms: Multi-task MS and LVEF classification, Transferred multi-task MS and LVEF classification, and Single-task MS and LVEF classification.

### Training and evaluation strategy

All models were trained on an NVIDIA RTX3080Ti Graphics Processing Unit (NVIDIA, Santa Clara, CA, USA). The training strategy is detailed in the Supplementary Methods. We evaluated the performance of all models using the old- and/or new-format test sets in MS and LVEF < 50% classification. We also compare the performance between the models trained on ECG images and the ones trained on the extracted signals. We compared the area under the receiver operating curves (AUCs) of the fully trained models on each test dataset against the baseline. To account for the presence of class imbalances, their F1 scores were also evaluated. Since we aim to develop a model that could be deployed for screening purposes, we also assessed the true positive rate (TPR) and false positive rates (FPR) for each model at different probability threshold levels.

To compare the performance of our models with the cardiologists, we recruited two cardiologists—one experienced and one in training. We asked the two cardiologists to independently review and interpret ECGs from new-format test datasets. The details of the interpretation process are in the Supplementary Methods. AUC, sensitivity, specificity, and F1 score were the diagnostic performance parameters used as evaluation metrics.

### Statistical analysis

All statistical analyses were performed using Python, version 3.8.13 (Python Software Foundation, Wilmington, DE, USA) and Medcalc, version 20.100 (MedCalc Software Ltd., Ostend, Belgium). The scikit-learn library (scikit-learn, Paris, France)^43^ was used for evaluation metrics calculation. A two-sided 95% confidence interval (95%CI) of AUC values was estimated using the binomial exact method^44^.

### Localization of models’ decision

To understand the rationale behind the model’s prediction, we employed “Grad-CAM++”, a technique to visualize the activation map for CNN^45^. This technique generated a heatmap that highlights the pertinent region in the ECG image that significantly contributes to the prediction of the model. Red areas indicated higher attention from the model, while blue areas indicated less contribution to the prediction.

### Sensitivity analysis

#### Prevalence-specific analysis

The reported prevalence of MS was estimated to be 7.9% in the general US population aged 45–84 years with no clinical cardiovascular disease^46^. We evaluated the performance of our model using a new-format test set with a case–control ratio matching a prevalence of MS of 8.0%. We applied bootstrap sampling to simulate a screening program in a normal population. This was achieved by randomly selecting 460 cases and 40 controls from the test dataset 1000 times to estimate the AUC (Fig. 1a).

#### Performance in detecting LVEF < 40%

We evaluated the performance of the models trained to predict LVEF < 50% and then used them to predict LVEF < 40% in the test population. This 40% LVEF cut point was selected to evaluate the performance of the models for detecting patients with heart failure with reduced ejection fraction.

## Conclusion

The results of this study demonstrate the potential of computer-based MS and LVEF classification using ECG scan images in clinical screening context. Implementing this prediction model would reduce costs, decrease dependence on CMR, facilitate care in areas with a shortage of cardiologists, and accelerate the treatment initiation or referral in identified cases. These results lay the groundwork for improved CAD screening in Thailand and in other limited-resource settings worldwide. Further study is needed to explore the model's applicability to other cardiovascular diseases.

## Supplementary Information

Supplementary Information.

## Data Availability

All datasets generated for this study are available from the corresponding author upon reasonable request. Requests to access these datasets should be directed to Prof. Dr. Rungroj Krittayaphong at rungroj.kri@mahidol.ac.th. All of the codes generated in this study are available at https://github.com/biodatlab/multitask-vision-ecg.

## References

[1] GBD 2013 Mortality and Causes of Death Collaborators. Global, regional, and national age-sex specific all-cause and cause-specific mortality for 240 causes of death, 1990–2013: A systematic analysis for the Global Burden of Disease Study 2013. *Lancet* **385**, 117–171 (2015). 25530442 10.1016/S0140-6736(14)61682-2 PMC4340604

[2] Ralapanawa, U. & Sivakanesan, R. Epidemiology and the magnitude of coronary artery disease and acute coronary syndrome: A narrative review. *J. Epidemiol. Glob. Health* **11**, 169–177 (2021). 33605111 10.2991/jegh.k.201217.001 PMC8242111

[3] Arenja, N. *et al.* Prevalence, extent, and independent predictors of silent myocardial infarction. *Am. J. Med.* **126**, 515–522 (2013). 23597799 10.1016/j.amjmed.2012.11.028

[4] Ponikowski, P. *et al.* Heart failure: Preventing disease and death worldwide. *ESC Heart Fail.* **1**, 4–25 (2014). 28834669 10.1002/ehf2.12005

[5] Groenewegen, A., Rutten, F. H., Mosterd, A. & Hoes, A. W. Epidemiology of heart failure. *Eur. J. Heart Fail.* **22**, 1342–1356 (2020). 32483830 10.1002/ejhf.1858 PMC7540043

[6] Callender, T. *et al.* Heart failure care in low- and middle-income countries: A systematic review and meta-analysis. *PLoS Med.* **11**, e1001699 (2014). 25117081 10.1371/journal.pmed.1001699 PMC4130667

[7] Heidenreich, P. A. *et al.* 2022 AHA/ACC/HFSA guideline for the management of heart failure: A report of the American college of cardiology/American heart association joint committee on clinical practice guidelines. *Circulation* **145**, e895–e1032 (2022). 35363499 10.1161/CIR.0000000000001063

[8] McDonagh, T. A. *et al.* 2021 ESC guidelines for the diagnosis and treatment of acute and chronic heart failure. *Eur. Heart J.* **42**, 3599–3726 (2021). 34447992 10.1093/eurheartj/ehab368

[9] Collet, J.-P. *et al.* 2020 ESC guidelines for the management of acute coronary syndromes in patients presenting without persistent ST-segment elevation. *Rev. Esp. Cardiol.* **74**, 544 (2021). 34020768 10.1016/j.rec.2021.05.002

[10] Kim, R. J. *et al.* The use of contrast-enhanced magnetic resonance imaging to identify reversible myocardial dysfunction. *N. Engl. J. Med.* **343**, 1445–1453 (2000). 11078769 10.1056/NEJM200011163432003

[11] Richardson, W. J., Clarke, S. A., Quinn, T. A. & Holmes, J. W. Physiological implications of myocardial scar structure. *Compr. Physiol.* **5**, 1877–1909 (2015). 26426470 10.1002/cphy.c140067 PMC4727398

[12] Hongo, R. H. & Goldschlager, N. Status of computerized electrocardiography. *Cardiol. Clin.* **24**(491–504), x (2006). 16939838 10.1016/j.ccl.2006.03.005

[13] Reichlin, T. *et al.* Advanced ECG in 2016: Is there more than just a tracing?. *Swiss Med. Wkly.* **146**, w14303 (2016). 27124801 10.4414/smw.2016.14303

[14] Jentzer, J. C. *et al.* Left ventricular systolic dysfunction identification using artificial intelligence-augmented electrocardiogram in cardiac intensive care unit patients. *Int. J. Cardiol.* **326**, 114–123 (2021). 33152415 10.1016/j.ijcard.2020.10.074

[15] Jeong, D. U. & Lim, K. M. Convolutional neural network for classification of eight types of arrhythmia using 2D time-frequency feature map from standard 12-lead electrocardiogram. *Sci. Rep.* **11**, 20396 (2021). 34650175 10.1038/s41598-021-99975-6 PMC8516863

[16] Hughes, J. W. *et al.* Performance of a convolutional neural network and explainability technique for 12-lead electrocardiogram interpretation. *JAMA Cardiol.* **6**, 1285–1295 (2021). 34347007 10.1001/jamacardio.2021.2746 PMC8340011

[17] Vaid, A. *et al.* Using deep-learning algorithms to simultaneously identify right and left ventricular dysfunction from the electrocardiogram. *JACC Cardiovasc. Imaging* **15**, 395–410 (2022). 34656465 10.1016/j.jcmg.2021.08.004 PMC8917975

[18] Ma, P., Li, Q. & Li, J. Application of artificial intelligence in cardiovascular imaging. *J. Healthc. Eng.* **2022**, 7988880 (2022). 35070243 10.1155/2022/7988880 PMC8769830

[19] Dima, S.-M. *et al.* On the detection of myocadial scar based on ECG/VCG analysis. *IEEE Trans. Biomed. Eng.* **60**, 3399–3409 (2013). 24001951 10.1109/TBME.2013.2279998

[20] Gumpfer, N., Grün, D., Hannig, J., Keller, T. & Guckert, M. Detecting myocardial scar using electrocardiogram data and deep neural networks. *Biol. Chem.* **402**, 911–923 (2021). 33006947 10.1515/hsz-2020-0169

[21] Perez Alday, E. A. *et al.* Classification of 12-lead ECGs: The physionet/computing in cardiology challenge 2020. *Physiol. Meas.* **41**, 124003 (2021). 33176294 10.1088/1361-6579/abc960 PMC8015789

[22] Wu, Y., Yang, F., Liu, Y., Zha, X. & Yuan, S. *A Comparison of 1-D and 2-D Deep Convolutional Neural Networks in ECG Classification*. arXiv [cs.CV] (2018).

[23] Wagner, P. *et al.* PTB-XL, a large publicly available electrocardiography dataset. *Sci. Data* **7**, 154 (2020). 32451379 10.1038/s41597-020-0495-6 PMC7248071

[24] Xiong, P., Lee, S.M.-Y. & Chan, G. Deep learning for detecting and locating myocardial infarction by electrocardiogram: A literature review. *Front. Cardiovasc. Med.* **9**, 860032 (2022). 35402563 10.3389/fcvm.2022.860032 PMC8990170

[25] Ansari, S. *et al.* A review of automated methods for detection of myocardial ischemia and infarction using electrocardiogram and electronic health records. *IEEE Rev. Biomed. Eng.* **10**, 264–298 (2017). 29035225 10.1109/RBME.2017.2757953 PMC9044695

[26] Hao, P. *et al.* Multi-branch fusion network for myocardial infarction screening from 12-lead ECG images. *Comput. Methods Programs Biomed.* **184**, 105286 (2020). 31891901 10.1016/j.cmpb.2019.105286

[27] Reinier, K. *et al.* Electrical surrogate for detection of severe left ventricular systolic dysfunction. *Ann. Noninvasive Electrocardiol.* **23**, e12591 (2018). 30126010 10.1111/anec.12591 PMC6264892

[28] Swartz, M. H., Pichard, A. D., Meller, J., Teichholz, L. E. & Herman, M. V. The normal electrocardiogram as a predictor of left ventricular function in patients with coronary artery disease. *Br. Heart J.* **39**, 208–211 (1977). 836737 10.1136/hrt.39.2.208 PMC483218

[29] Vaid, A. *et al.* Automated determination of left ventricular function using electrocardiogram data in patients on maintenance hemodialysis. *Clin. J. Am. Soc. Nephrol.* **17**, 1017–1025 (2022). 35667835 10.2215/CJN.16481221 PMC9269621

[30] Sun, J.-Y. *et al.* A method to screen left ventricular dysfunction through ECG based on convolutional neural network. *J. Cardiovasc. Electrophysiol.* **32**, 1095–1102 (2021). 33565217 10.1111/jce.14936

[31] Attia, Z. I. *et al.* Screening for cardiac contractile dysfunction using an artificial intelligence-enabled electrocardiogram. *Nat. Med.* **25**, 70–74 (2019). 30617318 10.1038/s41591-018-0240-2

[32] Kramer, C. M. Role of cardiac MR imaging in cardiomyopathies. *J. Nucl. Med.* **56**(Suppl 4), 39S-45S (2015). 26033902 10.2967/jnumed.114.142729 PMC4465292

[33] Thygesen, K. *et al.* Fourth universal definition of myocardial infarction (2018). *Eur. Heart J.* **40**, 237–269 (2019). 30165617 10.1093/eurheartj/ehy462

[34] Buck, S. F. A method of estimation of missing values in multivariate data suitable for use with an electronic computer. *J. R. Stat. Soc. Ser. B Stat. Methodol.* **22**, 302–306 (1960).

[35] Tipping, M. E. *Sparse Bayesian Learning and the Relevance Vector Machine*. https://www.jmlr.org/papers/volume1/tipping01a/tipping01a.pdf (2001).

[36] Christov, I. I. Real time electrocardiogram QRS detection using combined adaptive threshold. *Biomed. Eng. Online* **3**, 28 (2004). 15333132 10.1186/1475-925X-3-28 PMC516783

[37] Carreiras, C. *et al.**BioSPPy**: **Biosignal Processing in Python*. https://github.com/PIA-Group/BioSPPy/ (2015-).

[38] He, K., Zhang, X., Ren, S. & Sun, J. *Deep Residual Learning for Image Recognition*. arXiv [cs.CV] (2015).

[39] Jing, E. *et al.* ECG heartbeat classification based on an improved ResNet-18 model. *Comput. Math. Methods Med.* **2021**, 6649970 (2021). 34007306 10.1155/2021/6649970 PMC8110414

[40] Li, J. *et al.* Two-dimensional ECG-based cardiac arrhythmia classification using DSE-ResNet. *Sci. Rep.* **12**, 14485 (2022). 36008568 10.1038/s41598-022-18664-0 PMC9411603

[41] Sakli, N. *et al.* ResNet-50 for 12-lead electrocardiogram automated diagnosis. *Comput. Intell. Neurosci.* **2022**, 7617551 (2022). 35528345 10.1155/2022/7617551 PMC9071921

[42] Chen, T. & Guestrin, C. XGBoost: A scalable tree boosting system. in *Proceedings of the 22nd ACM SIGKDD International Conference on Knowledge Discovery and Data Mining*, 785–794 (Association for Computing Machinery, 2016).

[43] Pedregosa, F. *et al.* Scikit-learn: Machine learning in Python. *J. Mach. Learn. Res.* **12**, 2825–2830 (2011).

[44] Clopper, C. J. & Pearson, E. S. The use of confidence or fiducial limits illustrated in the case of the binomial. *Biometrika* **26**, 404–413 (1934).

[45] Chattopadhay, A., Sarkar, A., Howlader, P. & Balasubramanian, V. N. Grad-CAM++: Generalized gradient-based visual explanations for deep convolutional networks. in *2018 IEEE Winter Conference on Applications of Computer Vision (WACV)*, 839–847 (2018).

[46] Turkbey, E. B. *et al.* Prevalence and correlates of myocardial scar in a US cohort. *JAMA* **314**, 1945–1954 (2015). 26547466 10.1001/jama.2015.14849 PMC4774246

[47] Gildenblat, J. & Contributors. *PyTorch Library for CAM Methods*. https://github.com/jacobgil/pytorch-grad-cam (2021).

