# Correction: Myocardial scar and left ventricular ejection fraction classification for electrocardiography image using multi-task deep learning

**DOI:** 10.1038/s41598-025-18185-6

**Published:** 2025-09-22

**Authors:** Atirut Boribalburephan, Sukrit Treewaree, Noppawat Tantisiriwat, Ahthit Yindeengam, Titipat Achakulvisut, Rungroj Krittayaphong

**Affiliations:** 1 Department of Biomedical Engineering, Faculty of Engineering, Mahidol University, Nakhon Pathom, Thailand; 2 Looloo Technology, Bangkok, Thailand; 3 Division of Cardiology, Department of Medicine, Faculty of Medicine Siriraj Hospital, Mahidol University, 2 Wanglang Road, Bangkoknoi, Bangkok, 10700 Thailand; 4 Her Majesty Cardiac Center, Faculty of Medicine Siriraj Hospital, Mahidol University, Bangkok, Thailand

Correction to: *Scientific Reports* 10.1038/s41598-024-58131-6, published online 29 March 2024

The original version of this Article contained an error in the Methods section, under the subheading ‘Study population’.

“The protocol for this study was approved by the Siriraj Institutional Review Board (SIRB), in accordance with the Declaration of Helsinki (MU-MOU CoA no. 46912022).”

now reads:

“The protocol for this study was approved by the Siriraj Institutional Review Board (SIRB), in accordance with the Declaration of Helsinki (MU-MOU CoA no. 469/2022).”

The original Article has been corrected.

