# Supplementary material for: Myocardial scar and left ventricular ejection fraction classification for electrocardiography image using multi-task deep learning
Source: Sci Rep. 2024 Mar 29;14:7523. doi: 10.1038/s41598-024-58131-6 (PMC10980683; doi:10.1038/s41598-024-58131-6)
Supplement: Supplementary file 1 — Supplementary Information. [file 41598_2024_58131_MOESM1_ESM.pdf]

Supplementary Information for Myocardial scar and left ventricular ejection fraction classification for electrocardiography image using multi-task deep learning

Atirut Boribalburephan, Sukrit Treewaree, Noppawat Tantisiriwat, Ahthit Yindeengam, Titipat Achakulvisut, Rungroj Krittayaphong

Table of Contents

Supplementary Methods……………………………………………………………………………………1

Cardiac magnetic resonance imaging protocols & parameters……………………………………1

ECG data preprocessing……………………………………………………………………………1

ECG Dataset splitting…………………………………………………………………………………2

Model architecture……………………………………………………………………………………2

Incorporating clinical features into the models……………………………………………………2

Training strategy…………………………………………………………………………………………3

Interpretation process by cardiologists…………………………………………………………………3

Supplementary Results…………………………………………………………………………………4

Supplementary Data 1 - Model performance results compared to baseline prediction……………4

Supplementary Data 2 – 1D Model performance results compared to baseline prediction…………8

Supplementary Data 3 - AUC and F1 score from different cross-entropy loss ratio………………11

Supplementary Figure 1 ………………………………………………………………………………15

Supplementary Figure 2 ………………………………………………………………………………16

References……………………………………………………………………………………………17

Supplementary Methods

Cardiac Magnetic Resonance (CMR) imaging protocols & parameters

CMR imaging was performed using a Gyroscan NT Intera 1.5-Tesla Philips scanner (Philips Medical Systems, Best, the Netherlands) during 2009-2016, and using an Ingenia 3.0T MR system (Philips Medical Systems) during 2017-2021.

For the black blood axial plain images and steady-state free precession (SSFP) cine images, the parameters for functional images were 3.7/1.8/2 for repetition time/echo time/number of excitations, 390 × 312-mm fields of view, 256 × 240 matrix, 1.52 × 1.21 reconstruction pixel, 8-mm slice thickness, and 70° flip angle.

For Delayed-enhancement cardiovascular magnetic resonance (DE-CMR) images, a dose of 0.15 mmol per kg of body weight of gadolinium-based MRI contrast agent [gadoterate meglumine (Dotarem; Guerbet, Paris, France), gadobutrol (Gadovist; Bayer Healthcare, Leverkusen, Germany), or gadopentetate dimeglumine (Magnevist; Bayer Healthcare)] was injected intravenously. The inversion time was adjusted to the null normal myocardium. The viability study was assessed in the short-axis, 2-chamber, and 4-chamber views at 10 minutes after intravenous administration. The images were acquired using a 3-dimensional segmented gradient echo and inversion-recovery sequence with the following parameters: 1.25 ms echo time, 4.1 ms repetition time, 15° flip angle, 303 × 384-mm field of view, 240 × 256 matrix, 1.26 × 1.5 mm in-plane resolution, 8 mm slice thickness, and 1.5 sensitivity encoding factor.

Left ventricular ejection fraction (LVEF) was assessed using left ventricular end-systolic and end-diastolic volumes calculated from multiple-slice short-axis images. Delayed-enhancement cardiovascular magnetic resonance (DE-CMR) images were interpreted to determine the location and pattern of the myocardial scar (MS). CMR image analysis was performed on the ISP workstation (IntelliSpace Portal 9.0, Philips Healthcare, Best, the Netherlands). The 17-segment model, as recommended by the American Heart Association[1] and with the exception of segment 17, was used. Delayed-enhancement images were read while blinded to the ECG results.

Electrocardiogram (ECG) data preprocessing

We preprocessed raw portable document format (PDF) of full ECG reports into 12-lead ECG scan images using a process consisting of three main steps. First, we converted the PDF file into an image file for each ECG report using

the *pdf2image* library in Python.[2] We then isolated the region containing the ECG signals and removed the gridlines by filtering out shades of red in the new format. Finally – to normalize all of the ECGs, we rearranged the ECG leads into two vertical columns (6 rows × 2 columns). The first column contains lead I, II, III, aVR, aVL and aVF, while the second column contains V1, V2, V3, V4, V5, and V6 (Figure 1B). We also cropped the signal regions of the old-format ECGs to normalize the number of QRS pulses to approximately the same number of QRS pulses shown in the new-format ECGs. Both voltage scale and time scale are the same across the ECGs within the same format. However, scales are not the same between old and new formats. This might enable the models to not rely on the actual pixel size of the signal on images but focus on the relative size of signals within each format. The average processing times for old-format and new-format ECG are 0.389 and 0.514 seconds, respectively. Processing the new-format ECG takes slightly longer due to the presence of the gridlines and the following removal process. We note that this was measured on AMD Ryzen 9 Mobile 4900H Laptop CPUs.

Dataset splitting by the year of ECG acquisition

Old-format ECGs from 2009 to 2014 were used for training, while old-format ECGs from 2015 and 2016 were used as development and test sets, respectively. New-format ECGs from 2017, and 2019 to 2021 were used for training. New-format ECGs from 2018 to 2019 were used as a development set, and new-format ECGs from 2018 were used as a test set.

Model architecture

The model uses ResNet34d[3] as the core backbone architecture. We used the resized image of size 384 x 384 pixels with augmentation in various aspects, including blurring, brightness, and contrast. The intermediate representation has a size of 512, which is forwarded to two separate dense classification heads – one for the MS classification and the other for the LVEF range classification. We developed the models using the PyTorch framework (PyTorch Foundation, Wilmington, Delaware) and Python (Python Software Foundation, Beaverton, Oregon).

Incorporating clinical features into the models

This model processes the ECG image through the ResNet34d backbone and processes the clinical features using a recurrent neural network (RNN)-based backbone before using the output from both to predict MS and LVEF range.

Embedding layers and RNN layers were shown to boost classification performance when used together as a feature extractor[4], and outperformed methods relying on multilayer perceptrons (MLP). In this study, we used a bidirectional RNN (BRNN) to represent the clinical features and to concatenate them to the intermediate layer for prediction. Categorical clinical features are transformed into a 512-sized embedding vector via an embedding layer, and the only numerical feature (age) is normalized and concatenated to the embedding vector. The combined vector then migrates to the BRNN layer to create a summary vector (Figure 2D). ECG and clinical representations are then concatenated before predictions are made.

In addition, we trained a similar single-task model with both image and clinical features as inputs for each task (Figure 2E). This further helps verify the effectiveness of training the model in a multi-task manner over training two separate single-task models where clinical features are also utilized.

Training strategy

All of the models developed in this study were trained for 30 epochs using an AdamW optimizer[5] with an initial learning rate of 0.0005 that was scheduled to reduce linearly over the training steps. A simple early stopping strategy was also adopted in which the training terminates once the validation loss plateaus for five epochs. The transferred model was trained using old-format ECGs for ten epochs, after which it was transferred to learn using new-format ECGs for another ten epochs.

Interpretation process by cardiologists

One experienced and one in training cardiologists were recruited and independently reviewed and interpreted ECGs from new-format test datasets. The interpretation was based on criteria for prior or silent/unrecognized myocardial infarction[6] using the abnormal Q wave definition (Q or QS wave abnormality [Q/QS] [code 1.1, 1.2]) published in The Minnesota Code Manual of Electrocardiographic Findings.[7]

Supplementary Results

Supplementary Data 1. Model performance evaluation of myocardial scar (MS) classification and left ventricular ejection fraction (LVEF) <50% classification when using the old- and new-format test sets.

(**Abbreviations:** AUC, area under the curve; F1, defined as the harmonic mean of precision and recall; FNR, false negative rate**;** FPR, false positive rate)

Supplementary Table 1.1 Model performance evaluation of MS classification when using the old-format test set

Model	Old-format test set (N=895)						
	Accuracy	Sensitivity	Specificity	F1	AUC	FPR	FNR
**Baseline**	73.63	-	100.00	62.45	-	0.00	-
**XGBoost (Clinical Features)**	71.28	30.51	85.89	69.48	68.51	14.11	69.49
**Multi-task (old format)**	82.57	57.63	91.50	81.96	83.47	8.5	42.37
**Multi-task (transferred)**	83.13	**59.32**	91.65	82.58	82.39	8.35	**40.68**
**Single-task (both formats)**	83.58	44.49	**97.57**	81.59	82.28	**2.43**	55.51
**Multi-task (both formats)**	**83.69**	54.66	94.08	**82.71**	**83.82**	5.92	45.34
**Single-task with clinical features**	82.91	55.51	92.72	82.09	83.63	7.28	44.49
**Multi-task with clinical features**	83.46	52.12	94.69	82.29	83.16	5.31	47.88

Supplementary Table 1.2 Model performance evaluation of MS classification when using the new-format test set

Model	New-format test set (N=1,264)						
	Accuracy	Sensitivity	Specificity	F1	AUC	FPR	FNR
**Baseline**	73.97	-	100.00	62.90	-	0.00	-
**XGBoost (Clinical Features)**	69.22	40.73	79.25	69.21	68.17	20.75	59.27
**Multi-task (old format)**	78.64	40.43	92.09	76.86	78.40	7.91	59.57
**Multi-task (transferred)**	83.15	56.84	92.41	82.44	82.88	7.59	43.16
**Single-task (both formats)**	**84.49**	52.89	**95.61**	**83.31**	83.74	**4.39**	47.11
**Multi-task (both formats)**	83.15	50.76	94.55	81.91	81.10	5.45	49.24
**Single-task with clinical features**	83.07	48.63	95.19	81.63	82.14	4.81	51.37
**Multi-task with clinical features**	83.07	**59.88**	91.23	82.59	**84.06**	8.77	**40.12**

Supplementary Table 1.3 Model performance evaluation of LVEF <50% classification when using the old-format test set

Model	Old-format test set (N=895)						
	Accuracy	Sensitivity	Specificity	F1	AUC	FPR	FNR
**Baseline**	80.67	-	100.00	72.04	-	0.00	-
**XGBoost (Clinical Features)**	68.83	44.49	77.54	69.17	66.87	22.46	55.51
**Multi-task (old format)**	88.94	68.21	93.91	88.80	92.90	6.09	31.79
**Multi-task (transferred)**	88.72	67.05	93.91	88.55	91.70	6.09	32.95
**Single-task (both formats)**	89.39	64.74	95.29	89.03	93.58	**4.71**	35.26
**Multi-task (both formats)**	**90.39**	**75.14**	**94.04**	**90.39**	**93.91**	5.96	**24.86**
**Single-task with clinical features**	85.81	60.12	91.97	85.63	85.85	8.03	39.88
**Multi-task with clinical features**	89.50	73.99	93.21	89.54	93.63	6.79	26.01

Supplementary Table 1.4 Model performance evaluation of LVEF <50% classification when using the new-format test set

Model	New-format test set (N=1,264)						
	Accuracy	Sensitivity	Specificity	F1	AUC	FPR	FNR
**Baseline**	81.96	-	100.00	73.84	-	0.00	-
**XGBoost (Clinical Features)**	67.72	24.62	82.89	65.96	63.74	17.11	75.38
**Multi-task (old format)**	86.39	51.75	94.02	85.75	87.80	5.98	48.25
**Multi-task (transferred)**	**89.40**	60.53	95.75	88.92	92.08	4.25	39.47
**Single-task (both formats)**	**89.32**	59.21	95.95	88.78	91.77	4.05	40.79
**Multi-task (both formats)**	**89.24**	55.26	**96.72**	88.47	**93.07**	**3.28**	44.74
**Single-task with clinical features**	86.08	52.63	93.44	85.54	84.36	6.56	47.37
**Multi-task with clinical features**	**89.32**	**68.42**	93.92	**89.24**	92.40	6.08	**31.58**

Supplementary Data 2. 1D Model performance evaluation of myocardial scar (MS) classification and left ventricular ejection fraction (LVEF) <50% classification when using the old- and new-format test sets.

(**Abbreviations:** AUC, area under the curve; F1, defined as the harmonic mean of precision and recall; FNR, false negative rate**;** FPR, false positive rate)

Supplementary Table 2.1 1D Model performance evaluation of MS classification when using the old-format test set

Model	Old-format test set (N=895)						
	Accuracy	Sensitivity	Specificity	F1	AUC	FPR	FNR
**Baseline**	73.63	-	100.00	62.45	-	0.00	-
**Multi-task (old format)**	78.10	**54.24**	86.65	77.78	76.86	13.35	45.76
**Multi-task (transferred)**	72.62	26.27	89.23	69.80	59.89	10.77	73.73
**Single-task (both formats)**	81.00	52.54	91.20	80.15	79.06	8.80	**47.46**
**Multi-task (both formats)**	**84.13**	50.00	**96.36**	**82.69**	**80.14**	**3.64**	50.00

Supplementary Table 2.2 1D Model performance evaluation of MS classification when using the new-format test set

Model	New-format test set (N=1263)						
	Accuracy	Sensitivity	Specificity	F1	AUC	FPR	FNR
**Baseline**	73.95	-	100.00	62.88	-	0.00	-
**Multi-task (old format)**	75.14	24.32	93.04	71.43	63.29	6.96	75.68
**Multi-task (transferred)**	78.70	34.65	**94.22**	76.10	75.33	5.78	65.35
**Single-task (both formats)**	**80.20**	37.99	**95.07**	77.85	**77.51**	**4.92**	62.00
**Multi-task (both formats)**	**80.05**	**46.81**	91.75	**78.80**	**76.81**	5.24	**53.19**

Supplementary Table 2.3 1D Model performance evaluation of LVEF <50% classification when using the old-format test set

Model	Old-format test set (N=895)						
	Accuracy	Sensitivity	Specificity	F1	AUC	FPR	FNR
**Baseline**	80.67	-	100.00	72.04	-	0.00	-
**Multi-task (old format)**	85.25	60.69	91.13	85.18	88.13	8.86	39.31
**Multi-task (transferred)**	79.78	43.35	88.50	79.42	75.00	11.49	56.65
**Single-task (both formats)**	89.05	61.27	**95.71**	88.55	91.56	**4.29**	38.73
**Multi-task (both formats)**	**90.05**	**67.63**	**95.43**	**89.78**	**93.16**	**4.57**	**32.37**

Supplementary Table 2.4 1D Model performance evaluation of LVEF <50% classification when using the new-format test set

Model	New-format test set (N=1263)						
	Accuracy	Sensitivity	Specificity	F1	AUC	FPR	FNR
**Baseline**	81.95	-	100.00	73.82	-	0.00	-
**Multi-task (old format)**	82.18	27.19	**94.30**	79.89	68.51	5.70	72.81
**Multi-task (transferred)**	86.86	60.09	92.75	**86.67**	86.82	7.25	39.91
**Single-task (both formats)**	**87.81**	57.02	**94.59**	**87.31**	**89.41**	**5.41**	42.98
**Multi-task (both formats)**	85.91	**64.91**	90.53	86.11	**89.89**	9.47	**35.09**

Supplementary Data 3 - AUC and F1 score from different cross-entropy loss ratio

(**Abbreviations:** AUC, area under the curve; F1, F1 score defined as the harmonic mean of precision and recall)

F1 score

Supplementary Table 3.1 F1 score of MS classification when using different cross-entropy loss ratio (scar: LVEF) in the old-format test set

Model	Old-format test set (N=895)		
	F1 (50-50)	F1 (60-40)	F1 (70-30)
Baseline	62.45		
Multi-task (old format)	**81.96**	79.57	78.60
Multi-task (transferred)	**82.58**	81.03	75.68
Multi-task (both formats)	**82.71**	82.13	81.57
Multi-task with Clinical Features	**82.29**	81.45	81.99

Supplementary Table 3.2 F1 score of LVEF <50% classification when using different cross-entropy loss ratio (scar:LVEF) in the old-format test set

Model	Old-format test set (N=895)		
	F1 (50-50)	F1 (60-40)	F1 (70-30)
Baseline	72.04		
Multi-task (old format)	88.80	**90.35**	88.37
Multi-task (transferred)	**88.55**	86.07	85.92
Multi-task (both formats)	**90.39**	87.69	89.15
Multi-task with Clinical Features	**89.54**	89.25	89.30

Supplementary Table 3.3 F1 score of MS classification when using different cross-entropy loss ratio (scar:LVEF) in the new-format test set

Model	New-format test set (N=1,264)		
	F1 (50-50)	F1 (60-40)	F1 (70-30)
Baseline	62.9		
Multi-task (old format)	76.86	77.17	**78.07**
Multi-task (transferred)	**82.44**	82.31	79.66
Multi-task (both formats)	81.91	81.19	**83.02**
Multi-task with Clinical Features	**82.59**	82.43	82.43

Supplementary Table 3.4 F1 score of LVEF <50% classification when using different cross-entropy loss ratio (scar:LVEF) in the new-format test set

Model	New-format test set (N=1,264)		
	F1 (50-50)	F1 (60-40)	F1 (70-30)
Baseline	73.84		
Multi-task (old format)	85.75	**87.82**	87.41
Multi-task (transferred)	**88.92**	87.21	87.93
Multi-task (both formats)	88.47	**88.61**	89.16
Multi-task with Clinical Features	**89.24**	89.17	88.08

AUC

Supplementary Table 3.5 AUC of MS classification when using different cross-entropy loss ratio (scar:LVEF) in the old-format test set

**Model**	**Old-format test set (N=895)**		
	**AUC (50-50)**	**AUC (60-40)**	**AUC (70-30)**
Multi-task (old format)	**83.47**	79.13	78.62
Multi-task (transferred)	**82.39**	82.19	74.59
Multi-task (both formats)	**83.82**	79.51	83.41
Multi-task with Clinical Features	83.16	83.66	**84.77**

Supplementary Table 3.6 AUC of LVEF <50% classification when using different cross-entropy loss ratio (scar:LVEF) in the old-format test set

**Model**	**Old-format test set (N=895)**		
	**AUC (50-50)**	**AUC (60-40)**	**AUC (70-30)**
Multi-task (old format)	**92.90**	92.48	92.34
Multi-task (transferred)	**91.70**	89.73	89.19
Multi-task (both formats)	**93.91**	92.27	93.46
Multi-task with Clinical Features	93.63	93.07	**93.92**

Supplementary Table 3.7 AUC of MS classification when using different cross-entropy loss ratio (scar:LVEF) in the new-format test set

Model	New-format test set (N=1,264)		
	AUC (50-50)	AUC (60-40)	AUC (70-30)
Multi-task (old format)	**78.40**	76.19	77.24
Multi-task (transferred)	**82.88**	81.98	78.94
Multi-task (both formats)	81.10	80.95	**83.65**
Multi-task with Clinical Features	84.06	83.99	**84.07**

Supplementary Table 3.8 AUC of LVEF <50% classification when using different cross-entropy loss ratio (scar:LVEF) in the new-format test set

Model	New-format test set (N=1,264)		
	AUC (50-50)	AUC (60-40)	AUC (70-30)
Multi-task (old format)	87.80	**89.43**	87.74
Multi-task (transferred)	**92.08**	90.62	89.47
Multi-task (both formats)	**93.07**	91.08	91.66
Multi-task with Clinical Features	92.40	**92.81**	91.64

Supplementary Figure 1.

ROC curves show the specificity and sensitivity of MS classification compared between experienced and in-training cardiologists. The AUC was quite similar for both sensitivity and specificity when compared between experienced and in-training cardiologists. The overall AUC for experienced cardiologists and in-training cardiologists was 0.683 and 0.654, respectively.

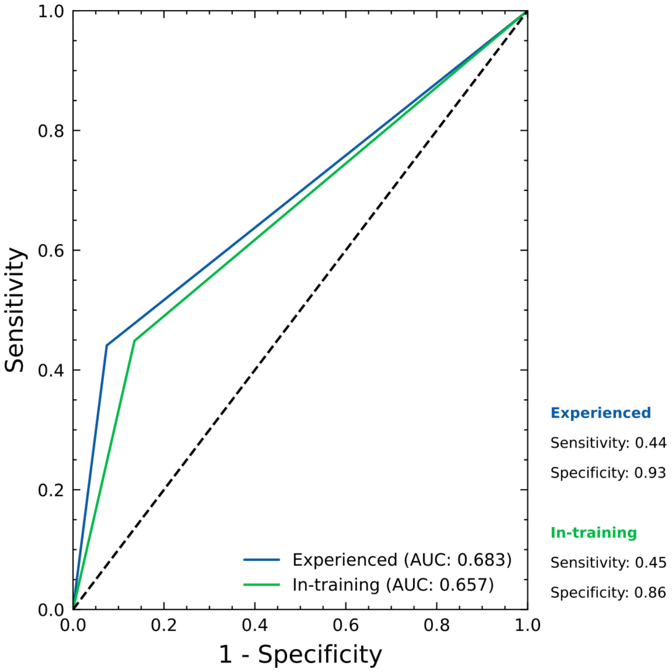

.

(**Abbreviations:** AUC, area under the ROC curve; MS, myocardial scar; ROC, receiver operating characteristic)

Supplementary Figure 2.

ROC curves showing the specificity and sensitivity of LVEF <40% classification for each evaluated model when using the old-format **(A)** and new-format **(B)** test sets. The AUC compared between the old-format and new-format test sets was quite similar for all models.

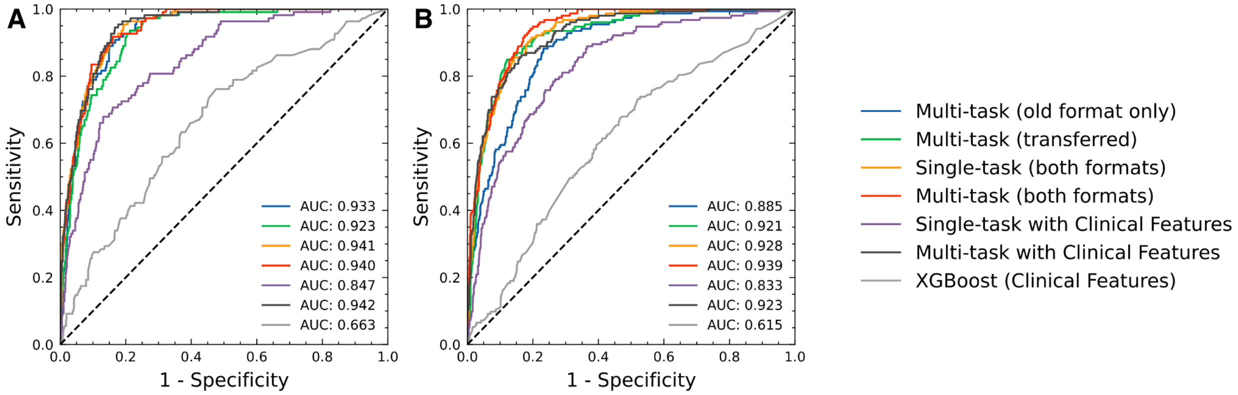

(**Abbreviations:** AUC, area under the ROC curve; LVEF, left ventricular ejection fraction; ROC, receiver operating characteristic)

References

1 Cerqueira, M. D. *et al.* Standardized Myocardial Segmentation and Nomenclature for Tomographic Imaging of the Heart. *Circulation* **105**, 539-542 (2002). https://doi.org:10.1161/hc0402.102975

2 Beval, E. *pdf2image*, <https://pypi.org/project/pdf2image/> (2017).

3 He, K., Zhang, X., Ren, S. & Sun, J. Deep Residual Learning for Image Recognition. *arXiv pre-print server* (2015). https://doi.org:10.48550/arXiv.1512.03385

4 Dahouda, M. K. & Joe, I. A Deep-Learned Embedding Technique for Categorical Features Encoding. *IEEE Access* **9**, 114381-114391 (2021). https://doi.org:10.1109/ACCESS.2021.3104357

5 Loshchilov, I. & Hutter, F. Decoupled Weight Decay Regularization. *arXiv pre-print server* (2019). https://doi.org:10.48550/arXiv.1711.05101

6 Thygesen, K. *et al.* Fourth Universal Definition of Myocardial Infarction (2018). *Circulation* **138**, e618-e651 (2018). https://doi.org:10.1161/CIR.0000000000000617

7 Prineas, R. J., Crow, R. S. & Zhang, Z.-M. *The Minnesota code manual of electrocardiographic findings*. (Springer Science & Business Media, 2009).
